# To what extent do people living with HIV, people on pre-exposure prophylaxis, doctors and pharmacists endorse 90-day dispensing of antiretroviral therapy in France?

**DOI:** 10.1371/journal.pone.0265166

**Published:** 2022-04-08

**Authors:** Christine Jacomet, Émilie Goncalves, Céline Lambert, Didier Chedorge, Sylvia Puglièse-Wehrlen, Éric Billaud, David Zucman, Anne Simon, Cédric Arvieux, Hervé Trout, Bruno Laurandin, René Maarek, Isabelle Raymond, Pascal Puglièse, Julie Langlois, Agnès Certain

**Affiliations:** 1 Infectious Diseases Department, COREVIH Auvergne Loire, Clermont-Ferrand University Hospital, Clermont-Ferrand, France; 2 Biostatistics Unit (DRCI), Clermont-Ferrand University Hospital, Clermont-Ferrand, France; 3 Pharmacist, Lyon, France; 4 Pharmaceutical Department, Nice University Hospital, Université Côte d’Azur, Nice, France; 5 Infectious Diseases Department, COREVIH Pays de la Loire, CIC 1413 INSERM, Nantes University Hospital, Nantes, France; 6 Internal Medicine Department, Foch Hospital, Suresnes, France; 7 Internal Medicine Department, Pitié Salpêtrière University Hospital, APHP, Paris, France; 8 Infectious Diseases Department, COREVIH-Bretagne, Rennes University Hospital, Rennes, France; 9 Pharmaceutical Department, Lariboisière Saint Louis and Fernand-Widal University Hospital, APHP, Paris, France; 10 Pharmacist, Suresnes, France; 11 Pharmacist, Montreuil, France; 12 Pharmaceutical Department, Bordeaux University Hospital, Bordeaux, France; 13 Infectious Diseases Department, Nice University Hospital, Université Côte d’Azur, Nice, France; 14 Société Française de Lutte Contre le SIDA, Nice, France; 15 Infectious Diseases Department, Bichat-Claude Bernard University Hospital, APHP, Paris, France; University of North Carolina at Chapel Hill, UNITED STATES

## Abstract

**Justification:**

The WHO 95-95-95 targets for 2030 do not imply that people living with HIV (PLHIV) achieve a good quality of life. The current 30-day dispensing interval for antiretroviral (ART) burdens the healthcare system. Lengthening dispensing intervals could alleviate this burden as well as enhance patient well-being.

**Objectives:**

To capture perceptions on 90-day dispensing interval (90D) for ART from the perspective of PLHIV, people on pre-exposure prophylaxis (PrEP), doctors, and pharmacists.

**Methods:**

Multi-centre observational survey led in France from 16 to 20 October 2020, among doctors agreeing to participate via regional coordinated care organisations for HIV, all PLHIV or people on PrEP consulting these outpatient-clinic doctors, and pharmacists doing ART dispensing.

**Results:**

The survey was completed by 220 doctors who saw 1087 people (999 PLHIV; 88 on PrEP) and 176 pharmacists from 55 centres. Among the PLHIV, 855 (85.6%, 95% CI: 83.2%–87.7%) and among the patients on PrEP, 70 (79.5%, 95% CI: 69.6%–87.4%) stated they would be interested in 90D. All in all, patients who were more likely to endorse 90D are those who opt exclusively for hospital dispensing (OR 3.22 [1.57–6.58]) and who rotate between hospital and community pharmacy dispensing (OR 3.29 [1.15–9.32]). Patients who were less likely to endorse 90-D were those who consult in a city located outside the 3 French high HIV prevalence regions (OR 0.66 [0.44–0.99]), receive 2 *vs* 1 pill QD regimens (OR 0.53 [0.31–0.91]), and anticipate at least one *vs* no limitation to 90D (OR 0.27 [0.17–0.42]). 90D was perceived as possible by 152 pharmacists (86.4%), including 8 (5%) without restriction, and 219 doctors (99.6%), including 42 (19.2%) regardless of PLHIV’s immunovirologic status or social conditions (health insurance coverage, access to housing or accommodation, access to rights, resources). Comparison of the benefits and limitations of a 90-day ART dispensing interval as perceived by PLHIV and people on PrEP, doctors and pharmacists shows that doctors anticipate a higher number of benefits than people on ART and/or pharmacists, chiefly that 90D would be more convenient and create less risk of drug shortages and that patients would gain autonomy and a better quality of life. Pharmacists were found to clearly perceive the economic benefits (90D would be less expensive) but anticipate more drawbacks than doctors and the people on ART themselves: more administrative burdens, more non-dispensing if doses get lost, harder to track adherence and more drug–drug interaction issues, and more work as they shall have to warn the patient of potential risks of shortages due to the cost of the stock.

**Conclusion:**

A clear majority of PLHIV, people on PrEP, doctors, and pharmacists endorsed 90D of ART. Most patients thought that 90D would be a good option, whereas most pharmacists and doctors thought that eligibility for 90D dispensing should depend on immunovirologic factors and social condition criteria. Moreover, pharmacists thought it would be necessary to commit regulatory resources and a better follow-up on adherence and drug–drug interactions.

## Introduction

The UNAIDS/WHO has set a ‘95-95-95’ HIV care cascade strategy by 2030 that aims at 95% of all people living with HIV infection (PLHIV) to be diagnosed, 95% of all people with diagnosed HIV infection to be on antiretroviral therapy (ART), and 95% of all people on ART to have an undetectable viral load (viral suppression). The most recent national evaluation of the HIV care cascade in France, published in 2017, found that 97% of patients on ART under the French healthcare system had a viral load under the 200 copies/mL threshold [1], which means the ‘third 95’ target has been achieved. However, this care cascade still does not focus on quality of life. Quality of life can be affected by different factors such as poverty, migration, discrimination, and stigma towards HIV–and these circumstances can lead to difficulties in accessing health insurance coverage, housing or accommodation, and rights and/or resources [2]. Moreover, in France, the health system is generally seen as punishing and uninviting–a perception inherited from the ‘AIDS years’, including the obligation to come to the pharmacy every month to collect ART. An adaptation to the patient’s life by opting for the 90-day ART dispensing interval instead of the current 30-day interval, with only 4 ART refill appointments to schedule per year could be linked to a better quality of life.

Likewise, pre-exposure prophylaxis (PrEP) was approved in France in 2016 but has since been dispensed as a 30-day supply [3]. It could also be extended to a 90-day dispensing interval, especially for long-term-users whose life-long prevention must be enhanced. The link between prevention and quality of life should not be underestimated.

The multi-month option could reduce the work involved in dispensing ART, which might enable the health system to look after more patients living with HIV or not. The WHO now endorses 3–6 monthly refills of ART. Six-month dispensing is practiced in many countries, including Malawi [4, 5]. Moreover, 90-day or multi-month dispensing of ART is already practiced in other countries in Europe and some countries expanded 90D measures during the COVID-19 pandemic as a way to minimize risks for HIV patients during lockdowns by limiting the number of trips for ART refills [6, 7].

The main hindrance to 90D in France is regulatory constraints, with many health decision-making bodies (General Health Directorate, High Health Authority, National Health and Medicines Agency, National and Regional Health Insurance Funds). ART is currently dispensed as a 30-day (or 28-day) supply, except when patients are due to travel abroad and carry a statement to this effect signed by the prescriber; in that case they generally need advance approval from their local health insurance fund depending on health insurance coverage and sometimes the country of destination [8, 9].

There is already a framework for dispensing 3-month supplies of drugs for other chronic conditions, i.e. diabetes, hypertension, hypercholesterolemia, and osteoporosis, if the packs are appropriately sized and prove substantially more cost-effective [10]. Oral contraceptives already have 90-day-supply pack sizes. To associate HIV to other broader chronic care regimens for long-term patient centred care could have a strong social impact and enhance their quality of life. Furthermore, France could align with neighbouring countries such as Germany whose results have been quite positive [11].

ART dispensing volume needs careful consideration of several specific factors, namely the patient’s clinical and virological stability. Providing either 60 or 90-day dispensation during the first 6 months of ART treatment is currently being investigated as a realistic approach to improve care for patients whose treatments are to be properly assessed for tolerance and effectiveness in the first months of therapy.

The higher cost burden of modifying regimens due to failure in patient’s adherence, intolerance, toxicity for new ART initiations or other reasons, and the potential added risks of lost or damaged medication could be a concern but do not seem to play out in reality [4, 12]. In France, since nearly 95% of PLHIV had undetectable viral load six months after initiation of ART, thanks to therapeutic education programs developed over the years, and more recently to high adherence to new ARV “one pill a day” strategies and their lesser toxicity, the financial benefit ratio is evolving in favour of longer refills [13, 14]. To begin with, the net savings in our country would amount to 155 million (95% CI: 149.9 to 159.4 million) euros if ART in bulk packaging were widely used as soon as marketed, which seems compatible with the current proportion of stabilized HIV patients [15]. Thus, this financial risk–benefit ratio may argue in favour of dispensing a 90D supply could be greatly increased if we count add the lowering of costs due to the higher number of patients that can be managed by the same number of staff, the reduced use of transportation to the facility as well, as the reduction of time off work for patients to pick up their ART.

According to the coordinating French national insurance fund—*Caisse Nationale d’Assurance Maladie -*, the rule is that only pharmaceutical company-issued 90-day packs are reimbursable when dispensing a 90-day supply. The pharmaceutical companies would have to agree to standardize 90-day-supply pack sizes [16].

Pharmacists, who play an increasingly important role in HIV care delivery for PLHIV, assert that ART drug stock management will need to be carefully planned [17]. Pharmacies would need a broader inventory to cover the many two-drug and three-drug combination therapies on the market, and yet they could also end up reducing their stock inventory by ordering per-patient only.

This brief analysis summarizes the difficulty around gauging needs for HIV care and several regulatory, financial, technical, industrial, and logistical hurdles that need consideration for roll-out if 90D dispensing is found preferable to stakeholders. We set out to capture perceptions on the benefits and drawbacks of 90D ART delivery from the perspective of the key stakeholders groups of PLHIV, people taking PrEP, prescribing doctors, and dispensing pharmacists.

## Methods

We carried out a multi-centre observational survey in France from 16 to 20 October 2020 of doctors agreeing to participate via regional coordinated care organisations (COREVIH), of PLHIV or people on PrEP consulting these outpatient-clinic doctors, and of pharmacists who dispense ART. It is worth noting that this survey was carried out during the COVID-19 pandemic, which may have an impact on the perception of 90-Day dispensing as hospitals were focused exclusively on patients potentially exposed or infected by SARS-CoV-2, with access restrictions for other patients including PLHIV and people on PrEP. During this time, the national insurance fund authorized 2-month dispensing, but not more.

### Creation of surveys

A group of 14 doctors and pharmacists who are experts on ART designed survey drafts using their own practice and the bibliographical study available to this date [18–20]. The separate surveys for patients, doctors, and pharmacists were read and corrected by the TRT5 (an inter-associative group of associations fighting HIV, hepatitis, and STD around the challenges of clinical research and therapeutical progress in the defence of the patients’ interests). Four versions of the surveys were elaborated. The last versions were tested on 15 voluntary patients, doctors, and pharmacists for approval of quality, then corrected once again before finalization (S1–S6 Appendices).

### Conduct of investigation

Before the survey, a letter presenting the study was sent by the investigating centre to the 28 presidents of regional coordinated care organizations (COREVIH centres) in France and in overseas departments. In turn, they passed the information to doctors in charge of HIV and PrEP in local hospitals (S1 Appendix). If one of these doctors wished to participate in our study, he or she would send us an entry form and receive all the study documents, including the surveys (S1–S11 Appendices, S1 Protocol).

During the study week, after reading the information letter, the doctors of the participating centres responded (once) to the anonymised paper doctor survey and proposed to all PLHIV and people on PrEP who came for an appointment to answer (once) the anonymised paper patient survey (S3–S6 Appendices, S9 and S10 Appendices). The patients who went to collect their ART had been given the presentation and information letters (S9 and S11 Appendices) and the anonymised paper pharmacist survey (S7 and S8 Appendices). During the dispensation, the pharmacists would then fill the survey out (once) and send it back to the investigating centre.

The inclusion criteria was patients aged over 18 years old and on ART/PrEP for more than 6 months. Exclusion criteria were patients unable to complete the questionnaire, unable to speak French, and refusal to participate.

This observational study was designed to comply with the French research standard MR003 (health research without collection of consent), and was reviewed and approved by the ethics committee “CPP Sud-Est VI, Clermont-Ferrand” on June 17^th^ 2020 (Ref # 2020/CE39), and the protocol was in compliance with the French data protection agency CNIL (study #M200701) (S1 Protocol).

Concerning data security and safe storage, both the patients and practitioners completed a paper-copy anonymized file, and the clinical trials technician generated their anonymized personal data as an electronic case report form (eCRF) using a REDCap web app specifically created for this study [21, 22]. Access to the data was restricted exclusively to the Clinical Research Center—Clermont-Ferrand University Hospital responsible for data monitoring, security, privacy, and control.

Statistical analyses were performed using Stata software (version 15, StataCorp, College Station, US). All tests were two-sided with type I error set at 0.05. All variables were categorical and presented as frequencies and associated percentages. The rates of people, doctors, and pharmacists who positively endorsed 90D were expressed as percentages with 95% confidence intervals (CI). Comparisons between independent groups (endorsers vs non-endorsers, doctors vs pharmacists, etc.) were carried out by the Chi-square test or the Fisher’s exact test. Regarding the outcome ‘endorsement’, a multivariable analysis was implemented using a logistic regression, considering the covariates according to the bivariate results and clinical relevance. The results were expressed as odds-ratio (OR) and 95% CI.

This cross-sectional study conducted on a sample should make it possible to generalise the results to the entire target population. A margin of error on the estimate is defined to calculate the number of participants required, in addition to the expected proportion of the primary outcome. Thus, for an expected proportion of PLHIV interested in 90D of about 50%, the inclusion of at least 600 participants made it possible to obtain a precision of this proportion of ± 4% (S1 Protocol).

## Results

The survey was conducted via 55 care services across France and completed by 220 doctors, 1087 people who saw those doctors during the study period (999 PLHIV and 88 people taking PrEP), and by 176 pharmacists filling those prescriptions for ART or PrEP who agreed to take part in the survey.

### Descriptive characteristics of the sample population

Table 1 reports descriptive statistics on the demographics and medical characteristics of the people on ART which were surveyed. Among the 999 PLHIV taking ART, 654 (65.5%) were men, 552 (55.3%) had been on ART for more than 10 years, 687 (68.8%) were taking a one pill once-a-day therapy, 912 (91.3%) self-reported having an undetectable viral load, and 170 (17.0%) used hospital-only delivery for their ART. Among the 88 people taking PrEP, 100% were men, 59 (67.0%) had been on ART for more than 12 months, 59 (67.0%) were on a continuous regimen, and 4 (4.6%) used hospital-only delivery for their PrEP.

**Table 1:** Patients on ART—Demographics and medical characteristics.

		PLHIV on ART	Patients on PrEP
		(n = 999)	(n = 88)
**Gender**	Male	654 (65.5%)	88 (100.0%)
	Female	325 (32.5%)	0 (0.0%)
	Trans female	4 (0.4%)	0 (0.0%)
	Trans male	1 (0.1%)	0 (0.0%)
	Declined	1 (0.1%)	0 (0.0%)
	Missing data	14 (1.4%)	0 (0.0%)
**Care geography**	IDF PACA ARA^a^	404 (40.4%)	41 (46.6%)
	DOM	49 (4.9%)	4 (4.5%)
	Other regions	544 (54.5%)	43 (48.9%)
	Missing data	2 (0.2%)	0 (0.0%)
**Length of ART therapy**	< 1 year	34 (3.4%)	29 (33.0%)
	1–10 years	303 (30.3%)	59 (67.0%)
	>10 years	552 (55.3%)	0 (0.0%)
	Missing data	110 (11.0%)	0 (0.0%)
**Viral load**	Undetectable < 6 months	58 (5.8%)	N/A
	Undetectable > 6 months	854 (85.5%)	
	Detectable	59 (5.9%)	
	Missing data	28 (2.8%)	
**ART dispensing source**	Hospital	170 (17.0%)	4 (4.6%)
	Community pharmacy	751 (75.2%)	81 (92.0%)
	Both	73 (7.3%)	3 (3.4%)
	Missing data	5 (0.5%)	0 (0.0%)
**Type of ART dosing schedule**	1 pill a day	687 (68.8%)	N/A
	2 pills taken once a day	121 (12.1%)	
	3 pills taken once a day	123 (12.3%)	
	Pills taken more than once a day	64 (6.4%)	
	Missing data	4 (0.4%)	
**Treatment**	Daily	955 (95.6%)	59 (67.0%)
	Structured interrupted	35 (3.5%)	29 (33.0%)
	Missing data	9 (0.9%)	0 (0.0%)

ARA: Auvergne-Rhône-Alpes; ART: antiretroviral; DOM: French overseas; IDF: Île-de-France; N/A: does not apply; PACA: Provence-Alpes-Côte d’Azur.

^a^: IDF, PACA, ARA are region of high HIV prevalence in France.

Table 2 reports descriptive statistics on the 200 doctors and 176 pharmacists who took part in the survey. Of the doctors, 153 (69.5%) consulted in university hospitals, 114 (51.8%) had an active patient population of between 100 and 500 PLHIV, and 153 (69.6%) had an active patient population of fewer than 30 people on PrEP. Of the pharmacists, 137 (77.8%) had a community practice, 106 (60.2%) provided service to an active population of between 2 and 30 PLHIV, and 82 (46.6%) provided service to fewer than 2 people on PrEP. The doctor and pharmacist samples were significantly different in terms of location of respondents (more doctors in the French overseas departments and more pharmacists outside the HIV-prevalent Île-de-France/Provence-Alpes-Côte-d’Azur/Auvergne-Rhône-Alpes regions of mainland France; *p* = 0.046) and in terms of size of their active patient populations (more ‘small’ active HIV patient populations among doctors; *p*<0.001).

**Table 2:** Characteristics of the in-sample doctors and pharmacists.

			Doctors	Pharmacists
			(n = 220)	(n = 176)
**Region served**	IDF PACA ARA^a^		93 (42.3%)	71 (40.3%)
	DOM		13 (5.9%)	2 (1.1%)
	Other regions		112 (50.9%)	102 (58.0%)
	Missing data		2 (0.9%)	1 (0.6%)
**Practice setting**	University hospital		153 (69.5%)	24 (13.6%)
	General hospital		62 (28.2%)	11 (6.3%)
	Community practice		1 (0.5%)	137 (77.8%)
	Private hospital		0 (0.0%)	1 (0.6%)
	Missing data		4 (1.8%)	3 (1.7%)
**Size of active patient population of PLHIV**	Doctors^b^	<100	91 (41.4%)	NA
		100–500	114 (51.8%)	
		>500	12 (5.4%)	
		Missing data	3 (1.4%)	
	Pharmacists^b^	0–2	NA	25 (14.2%)
		2–30		106 (60.2%)
		>30		35 (19.9%)
		Missing data		10 (5.7%)
**Size of active patient population of PrEP takers**	Doctors	<30	153 (69.6%)	NA
		30–100	30 (13.6%)	
		>100	10 (4.5%)	
		Missing data	27 (12.3%)	
	Pharmacists	0–2	NA	82 (46.6%)
		2–30		42 (23.9%)
		>30		7 (4.0%)
		Missing data		45 (25.6%)

ARA: Auvergne-Rhône-Alpes; DOM: French overseas; IDF: Île-de-France; PACA: Provence-Alpes-Côte d’Azur; PrEP: pre-exposure prophylaxis; PLHIV: persons living with HIV.

^a^: IDF, PACA, ARA are regions of high HIV prevalence in France.

^b^: the prevalence of pharmacists in France (32/100 000 habitants) is much higher than the prevalence of doctors involved in HIV and PrEP in 2020 (around 1/100 000).

### 90-day ART dispensing as perceived by PLHIV and people on PrEP

Among the PLHIV, 855 (85.6%, 95% CI: 83.2%–87.7%) would be interested in 90D, 50 (5.0%) would not be interested, and 83 (8.3%) have no stated preference.

Among people on PrEP, 70 (79.5%, 95% CI: 69.6%–87.4%) would be interested in 90D, 3 (3.4%) would not be interested, and 13 (14.8%) have no stated preference.

**Fig 1:**
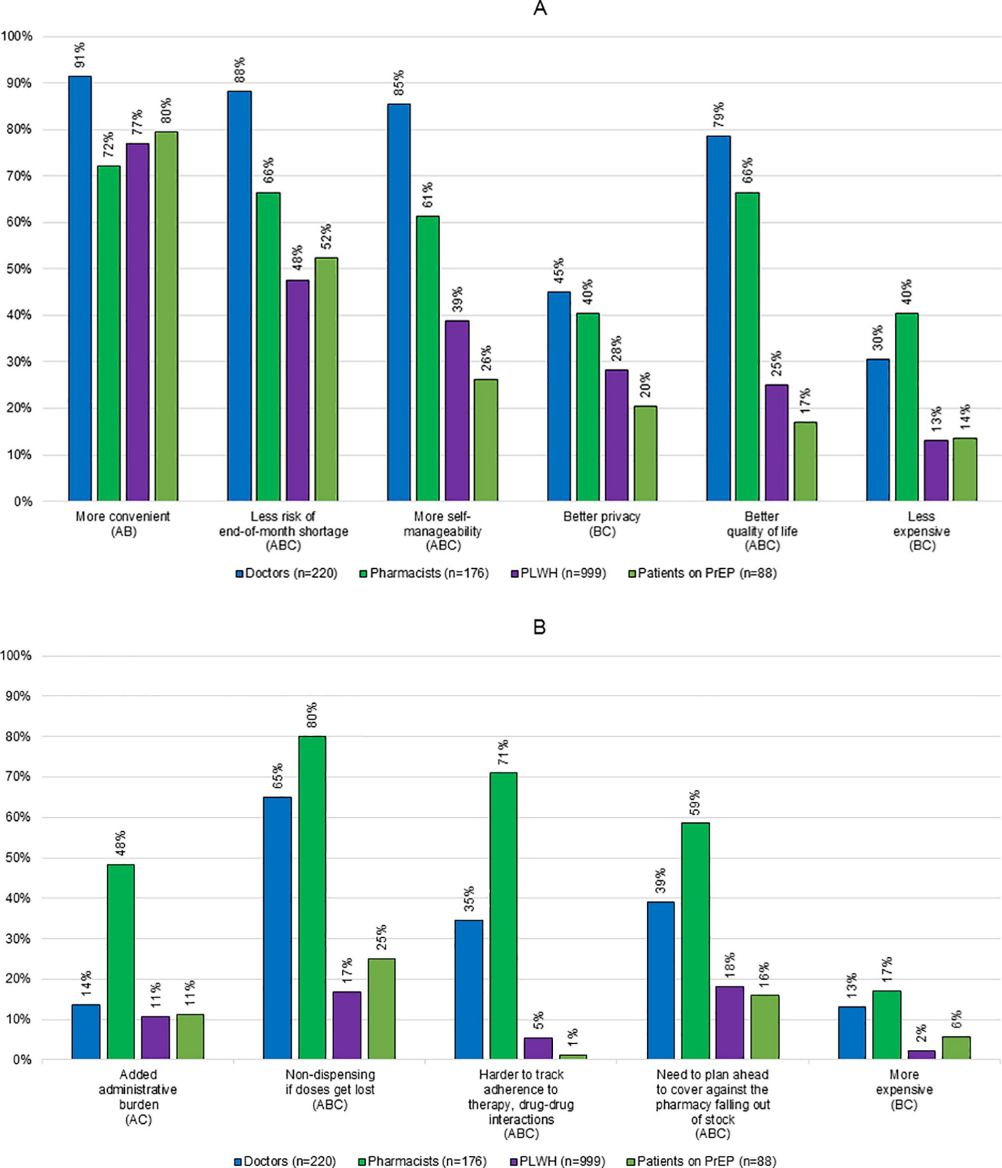
A. Comparison of the benefits of a 90-day ART dispensing interval reported by people on ART (PLHIV and people on PrEP) and their doctors and pharmacists. A = significant difference between doctors and pharmacists, B = significant difference between doctors and people on ART (PLHIV and people on PrEP), C = significant difference between pharmacists and people on ART (PLHIV and people on PrEP). B. Comparison of the limitations of a 90-day ART dispensing interval reported by people on ART (PLHIV and people on PrEP) and their doctors and pharmacists. A = significant difference between doctors and pharmacists, B = significant difference between doctors and people on ART (PLHIV and people on PrEP), C = significant difference between pharmacists and people on ART (PLHIV and people on PrEP).

The benefits of 90D dispensing (more convenient, less risk of end-of-month shortage, empowerment to self-manage, more confidential, better quality of life, less expensive) envisioned by PLHIV are on different scale than the ones envisioned by people on PrEP. The same goes for the limitations (risks of the pharmacy falling out of stock, anxiety over access to dispensing if a 90-day-supply pack gets lost, too much stock at home, risks of added regulatory complexities, insecurity around only seeing the pharmacist once every 3 months, more expensive) (Fig 1A and 1B).

**Fig 2:**
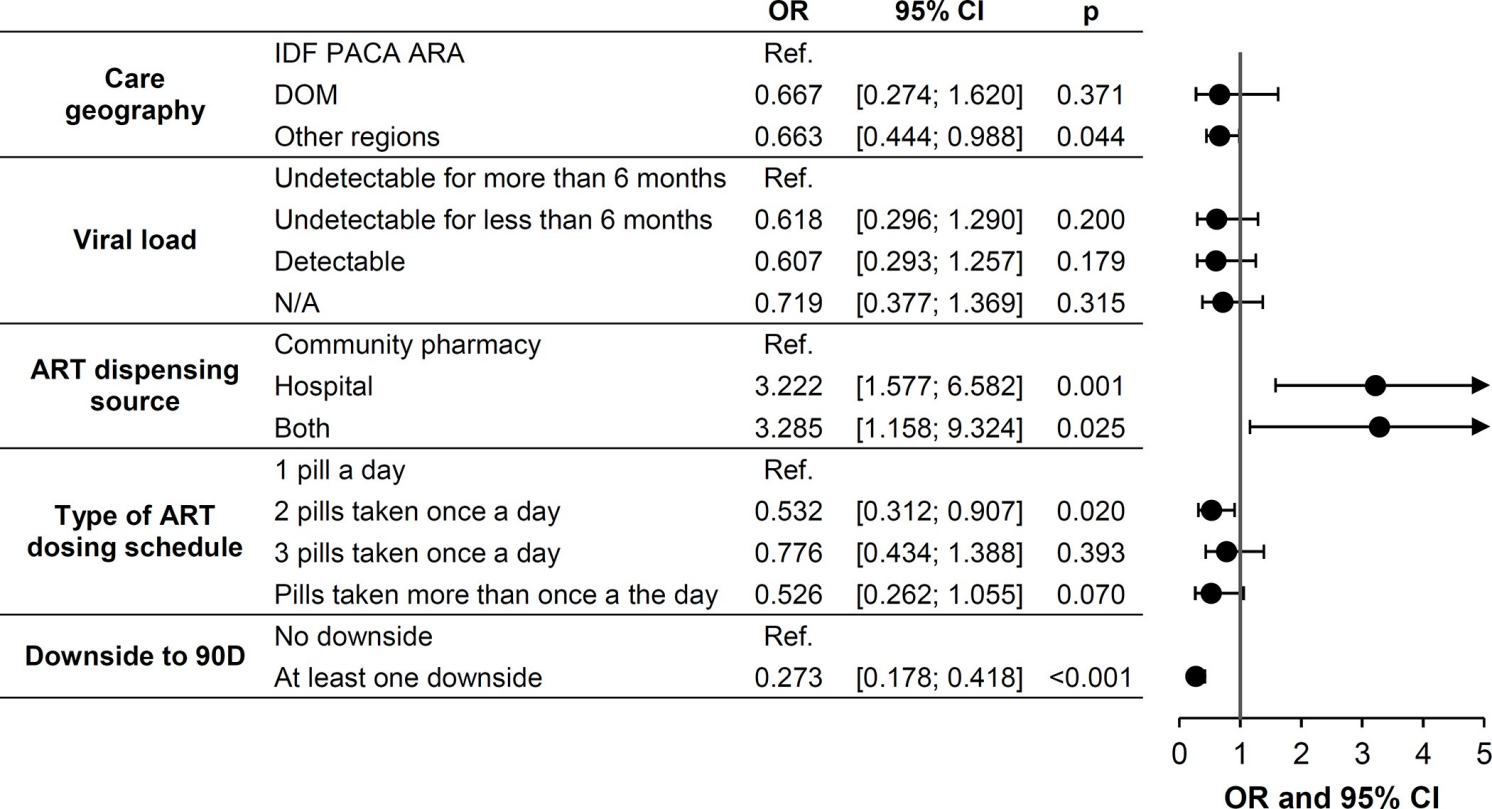
Characteristics of in-sample PLHIV and in-sample people on PrEP stratified as endorsers *vs* non-endorsers of 90-day ART dispensing (n = 1074). Multivariate analysis. ARA: Auvergne-Rhône-Alpes; ART: antiretroviral; DOM: French overseas; IDF: Île-de-France; N/A: does not apply; PACA: Provence-Alpes-Côte d’Azur. IDF, PACA, ARA are regions of high HIV prevalence in France.

Table 3 analyses the variables associated with PLHIV and patients on PrEP stratifying as 229 endorsers of 90-day ART dispensing versus non-endorsers or no preference (bivariate analyses). Multivariate analysis (Fig 2) found that the factors associated with non-endorsement of 90D by people on ART were attending in a city outside (*vs* inside) the Île-de-France/Provence-Alpes-Côte-D’Azur/Auvergne-Rhône-Alpes regions–high HIV prevalence regions–(OR 0.66 [0.44–0.99]), receiving a 2 pill *vs* 1 pill once-a-day regimen (OR 0.53 [0.31–0.91]), and anticipating at least one *vs* no limitation (OR 0.27 [0.17–0.42]). The factors associated with positive endorsement of 90D by people on ART were having opted for hospital dispensing of their ART either exclusively (OR 3.22 [1.57–6.58]) or in rotation with their community pharmacy (OR 3.29 [1.15–9.32]).

**Table 3:** Characteristics of in-sample PLHIV and in-sample people on PrEP stratified as endorsers *vs* non-endorsers of 90-day ART dispensing (n = 1074).

		Total PLHIV and people on PrEP	Endorsers	Non-endorsers^b^	*p*
		(n = 1074)	(n = 925)	(n = 149)	
**Gender**	Male	738 (68.7%)	629 (68.0%)	109 (73.1%)	0.21
	Female	323 (30.1%)	285 (30.8%)	38 (25.5%)	
	Trans female	4 (0.4%)	4 (0.4%)	0 (0.0%)	
	Trans male	1 (0.1%)	0 (0.0%)	1 (0.7%)	
	Refuses to self-identify	1 (0.1%)	1 (0.1%)	0 (0.0%)	
	Missing data	7 (0.6%)	6 (0.7%)	1 (0.7%)	
**Care geography**	IDF PACA ARA^a^	439 (40.9%)	391 (42.3%)	48 (32.2%)	0.004
	DOM	53 (4.9%)	46 (5.0%)	7 (4.7%)	
	Other regions	580 (54.0%)	488 (52.7%)	92 (61.7%)	
	Missing data	2 (0.2%)	0 (0.0%)	2 (1.4%)	
**Length of ART therapy**	< 1 year	62 (5.8%)	52 (5.6%)	10 (6.7%)	0.31
	1–10 years	358 (33.3%)	313 (33.8%)	45 (30.2%)	
	>10 years	547 (50.9%)	463 (50.1%)	84 (56.4%)	
	Missing data	107 (10.0%)	97 (10.5%)	10 (6.7%)	
**Viral load**	Undetectable < 6 months	58 (5.4%)	47 (5.1%)	11 (7.4%)	0.13
	Undetectable > 6 months	853 (79.4%)	746 (80.6%)	107 (71.8%)	
	Detectable	59 (5.5%)	47 (5.1%)	12 (8.1%)	
	N/A	86 (8.0%)	70 (7.6%)	16 (10.7%)	
	Missing data	18 (1.7%)	15 (1.6%)	3 (2.0%)	
**ART dispensing source**	Hospital	173 (16.1%)	164 (17.7%)	9 (6.0%)	<0.001
	Community pharmacy	821 (76.4%)	686 (74.2%)	135 (90.6%)	
	Both	75 (7.0%)	70 (7.6%)	5 (3.4%)	
	Missing data	5 (0.5%)	5 (0.5%)	0 (0.0%)	
**Type of ART dosing schedule**	1 pill a day	757 (70.5%)	669 (72.3%)	88 (59.1%)	0.004
	2 pills taken once a day	124 (11.5%)	99 (10.7%)	25 (16.8%)	
	3 pills taken once a day	123 (11.4%)	103 (11.2%)	20 (13.4%)	
	Pills taken more than once a day	63 (5.9%)	50 (5.4%)	13 (8.7%)	
	Missing data	7 (0.7%)	4 (0.4%)	3 (2.0%)	
**ART schedule**	Daily	1002 (93.3%)	865 (93.5%)	137 (91.9%)	0.70
	Structured interrupted	64 (6.0%)	53 (5.7%)	11 (7.4%)	
	Missing data	8 (0.7%)	7 (0.8%)	1 (0.7%)	
**Cited at least 1 upside to 90D**		1011 (94.1%)	922 (99.7%)	89 (59.7%)	<0.001
	More convenient	836 (82.7%)	793 (86.0%)	43 (48.3%)	<0.001
	Less risk of end-of-month shortages	519 (51.3%)	480 (52.1%)	39 (43.8%)	0.14
	More self-manageability	408 (40.4%)	391 (42.4%)	17 (19.1%)	<0.001
	Better quality of life	262 (25.9%)	256 (27.8%)	6 (6.7%)	<0.001
	Less expensive	141 (13.9%)	131 (14.2%)	10 (11.2%)	0.44
	More privacy	299 (29.6%)	287 (31.1%)	12 (13.5%)	<0.001
	Other	54 (5.3%)	50 (5.4%)	4 (4.5%)	1.00
**Cited at least one downside to 90D**		555 (51.7%)	440 (47.6%)	115 (77.2%)	<0.001
	Regulatory complexity	118 (21.3%)	106 (24.1%)	12 (10.4%)	0.001
	Anxiety over access to dispensing if doses get lost	190 (34.2%)	155 (35.2%)	35 (30.4%)	0.34
	Insecurity around less access to supportive services	55 (9.9%)	33 (7.5%)	22 (19.1%)	<0.001
	Risk of running out of stock	194 (35.0%)	169 (38.4%)	25 (21.7%)	0.001
	Too much stock at home	154 (27.7%)	82 (18.6%)	72 (62.6%)	<0.001
	More expensive	27 (4.9%)	22 (5.0%)	5 (4.3%)	0.77

ARA: Auvergne-Rhône-Alpes; ART: antiretroviral; DOM: French overseas; IDF: Île-de-France; N/A: does not apply; PACA: Provence-Alpes-Côte d’Azur; PrEP: pre-exposure prophylaxis; PLHIV: persons living with HIV.

^a^: IDF, PACA, ARA are regions of high HIV prevalence in France.

^b^: people who do not endorse a change in model or have no preference.

### 90-day ART dispensing as perceived by doctors and pharmacists

Among the 220 doctors surveyed, 219 (99.6%, 95% CI: 97.4%–100.0%) responded that dispensing patients a 90-day supply of ART in one pack would be possible, including 42 (19.2%) who thought it would be useful for all patients, not just for those with eligible therapeutic criteria (taking ART more than 6 months) but also for those with good immune and virological factors (CD4> 500/mm3 and undetectable viral load) and/or for those with favourable social conditions (having health insurance coverage, access to housing or accommodation, access to rights, sufficient resources). A univariate analysis showed that the doctors ready to endorse 90D for all patients *versus* only for patients with the above-mentioned criteria (Table 4) are those more often in charge of a great number of patients (p = 0.05).

**Table 4:** Characteristics of the 219 doctors endorsing 90-day dispensing of ART for all *vs* only for people with eligible therapeutic criteria and with good immune and virological factors and/or favourable social issues.

		Total	90-day dispensing for all	90-day dispensing for people with specific factors/issues	*p*
		(n = 219)	(n = 42)	(n = 177)	
**Region served**	IDF PACA ARA^a^	93 (42.5%)	18 (42.9%)	75 (42.4%)	0.93
	DOM	13 (5.9%)	3 (7.1%)	10 (5.6%)	
	Other regions	111 (50.7%)	21 (50.0%)	90 (50.9%)	
	Missing data	2 (0.9%)	0 (0.0%)	2 (1.1%)	
**Practice setting**	University hospital	152 (69.4%)	26 (61.9%)	126 (71.2%)	0.07
	General hospital	62 (28.3%)	13 (30.9%)	49 (27.7%)	
	Community practice	1 (0.5%)	1 (2.4%)	0 (0.0%)	
	Missing data	4 (1.8%)	2 (4.8%)	2 (1.1%)	
**Size of PLHIV population**	< 100	91 (41.5%)	13 (31.0%)	78 (44.1%)	0.005
	100–500	113 (51.6%)	22 (52.4%)	91 (51.4%)	
	> 500	12 (5.5%)	4 (9.5%)	8 (4.5%)	
	Missing data	3 (1.4%)	3 (7.1%)	0 (0.0%)	
**Size of PrEP population**	< 30	152 (69.4%)	31 (73.8%)	121 (68.4%)	0.22
	30–100	30 (13.7%)	2 (4.8%)	28 (15.8%)	
	> 100	10 (4.6%)	2 (4.8%)	8 (4.5%)	
	Missing data	27 (12.3%)	7 (16.7%)	20 (11.3%)	

ARA: Auvergne-Rhône-Alpes; DOM: French overseas; IDF: Île-de-France; PACA: Provence-Alpes-Côte d’Azur; PrEP: pre-exposure prophylaxis; PLHIV: persons living with HIV.

^a^: IDF, PACA, ARA are regions of high HIV prevalence in France.

Among the 176 pharmacists surveyed, 152 (86.4%, 95% CI: 80.3%–91.1%) thought that dispensing patients a 90-day supply of ART in one pack would be possible, including 8 (5.3%) who endorsed 90D without any restriction and 13 (8.6%) who endorsed 90D only if they had previously dispensed ART to that patient, whereas 14 pharmacists did not think it would be possible, 7 did not know, and 3 did not answer. A univariate analysis of the characteristics of pharmacists ready to endorse 90D *versus* others failed to reveal any defining characteristics.

Table 5 compares doctors and pharmacists in terms of differences in responses on criteria attached to 90D. A higher proportion of doctors than pharmacists endorses the transition to 90D regardless of treatment regimen (50.9% *vs* 31.3%, *p*<0.001), if therapy was initiated more than 6 months ago and there is an undetectable viral load (45.9% *vs* 26.1%, *p*<0.001), and if the patient’s social factors appear to be stable (45.0% *vs* 29.0%, *p* = 0.001). A higher proportion of pharmacists than doctors endorses the transition only for patients on a continuous regimen (37.5% *vs* 18.6%, *p*<0.001), regardless of the patient’s social factors (26.7% *vs* 15.9%, *p* = 0.008).

**Table 5:** Comparative analysis of responses from doctors and pharmacists concerning eligibility therapeutic, immunovirologic, or social conditions for 90-day ART dispensing.

	Doctors	Pharmacists	*p*
	(n = 220)	(n = 176)	
Positive endorsement of 90-day dispensing	219 (99.5%)	152 (86.4%)	<0.001
If the patient is a registered client^a^	N/A	13/152 (8.5%)	N/A
Without criteria	42/219 (19.2%)	8/152 (5.3%)	<0.001
**With criteria**	**177/219 (80.8%)**	**131/152 (86.2%)**	0.18
regardless of ART regimen	112 (50.9%)	55 (31.3%)	<0.001
only if therapy is one pill a day	6 (2.7%)	14 (8.0%)	0.02
if the therapy regimen is continuous	41 (18.6%)	66 (37.5%)	<0.001
if patient has been stable on the therapy more than 6 months	59 (26.8%)	52 (29.5%)	0.55
if viral load is undetectable and CD4 > 500	36 (16.4%)	17 (9.7%)	0.052
if adherence to therapy is good	52 (23.6%)	41 (23.3%)	0.94
if therapy was initiated more than 6 months ago and there is undetectable viral load	101 (45.9%)	46 (26.1%)	<0.001
regardless of the patient’s social factors^b^	35 (15.9%)	47 (26.7%)	0.008
if the patient’s social factors appear to be stable	99 (45.0%)	51 (29.0%)	0.001
on demand, regardless of the patient’s social factors	38 (17.3%)	20 (11.4%)	0.10

ART: antiretrovirals; N/A: does not apply.

^a^ This question was only put to pharmacists who were, in 2020, more numerous in France than doctors in charge of HIV and PrEP. A patient can go to any pharmacy to receive his or her ART, including a distant pharmacy to avoid stigmatization.

^b^ Social factors: housing, health insurance, resident permit.

### Comparison of the benefits and limitations of a 90D model as perceived by people on ART, doctors and pharmacists

A comparative analysis of the benefits and limitations (Figs 1 and 2) of a 90-day ART dispensing interval as perceived by people on ART (PLHIV and people taking PrEP) and their doctors and pharmacists showed that people on ART and doctors were more likely than pharmacists to mention benefits other than/including convenience and were less likely than pharmacists to mention drawbacks.

Doctors anticipated a higher number of benefits than people on ART and/or pharmacists, chiefly that 90D would be more convenient and create less risk of drug shortages and that patients would be more empowered to self-manage and gain a better quality of life.

Pharmacists were found to clearly perceive the economic benefits (90D would be less expensive) but anticipated more limitations than doctors and the people on ART themselves, including added administrative complexities, more non-dispensing if doses got lost, more difficulties in tracking adherence, more drug–drug interaction issues, and increased workload if they would need to warn patients of potential shortages due to the cost of stock.

## Discussion

This national-coverage survey reveals high rates of endorsement for 90D ART dispensing model in France in 2020 among PLHIV (85.6%), patients on PrEP (79.5%), doctors (99.6%) and pharmacists (86.4%). This high rate of endorsement appears linked to good outcomes with better ART adherence and tolerance in the last ten years due to new strategies that are better tolerated and just one pill a day combined with the implementation of therapeutic education within patient care structures, as demonstrated by the high undetectable viral load rate among PLHIV in France [13, 14]. Several studies published since 2014 have shown that there is no difference in terms of adherence to therapy when ART regimens are dispensed at longer intervals, including among the most adherence-resistant populations, i.e. teenagers, which further supports evidence that there is no loss of retention in ART, and ultimately no therapeutic failure when refill-to-refill interval is extended among stable patients [4, 5, 19, 20, 23].

The ‘random-week’ survey method as well as the demographic and medical characteristics of the PLHIV included suggest that the cohort is a representative sample, at least for people on ART, of the wider French HIV community. In the IPLESP [Pierre Louis Institute of Epidemiology and Public Health] report published on 22/09/2020 on indicators of care delivery to PLHIV in COREVIH centres, 64.9% of the PLHIV population in France in 2018 were men, and 91.49% of all PLHIV had a HIV viral load < 50 copies/mL [13]. The PLHIV included in this survey had similar characteristics. Furthermore, a study based on data extracted from the French national health information systems in 2018 found that 14.8% of PLHIV in the Pays de la Loire region had only been delivered ART through outpatient dispensing of hospital-reserved drugs [24], which is consistent with the patient rate found in our survey.

The many regions and places of practice of the doctors and pharmacists surveyed and the heterogeneity of their active populations of PLHIV and patients on PrEP reflect a broadly diverse HIV community and thus a broad diversity of opinion on 90D in France.

The main message of our survey results is that 85.6% of PLHIV and 79.5% people on PrEP would welcome 90D of ART. The lesser demand of 90D ART dispensing from people on PrEP is probably linked to the fact that, in France, nearly 49.5% of people on PrEP take prophylaxis on demand, so their rate of ART consumption is therefore reduced [25]. Note that the PrEP sample is not very representative as a small sample of only males; very few women are taking PrEP in France [26]. Positive endorsement of 90D was not linked to the type or the length of therapy, nor to cost issues, but to factors associated with the geography of HIV care delivery in the HIV-prevalent regions of mainland France, access to one pill once-a-day therapy, and hospital-only ART dispensing. These results corroborate that the 90D perceived benefits lead to more self-manageability, less end-of-month medication shortages, more privacy, and more convenience, enabling better quality of life with fewer trips to clinics and community centres. We believe these preferences need to be better taken into account and understood by policy makers, and that treatment delivery policy should be modified in favour of a multi-month dispensation therapy.

Policy change would require addressing systemic barriers such as the dispensing of 30D ARVs while strengthening support for PLHIV and PrEP patients. This would include the necessity to adapt support for all people on ART including new communication strategies to promote adherence. Such a change is more important as e-health is growing, improving access to information through health apps and messaging services such as those already used by PLHIV [27–29]. Healthcare workers are committed to using these new means of communication but they are aware of the need to comply with the rules of privacy and security. At this point, this progress allows PLHIV, people on PrEP, and their healthcare workers to keep in touch without having to meet in person. The COVID-19 pandemic has accelerated this trend.

All but one of the doctors surveyed here positively endorsed 90D, and 81% endorsed it with specific criteria that would need to be met before 90D could be used. Those criteria include the stability of the patient on ART for over 6 months which is in agreement with guidelines advocated in a recent paper in *The Lancet* [30], and with patients, who also felt that there needed to be criteria on who could receive longer refills [12]. This restriction was advocated by patients and healthcare workers, and across high- and lower-income settings. Other criteria highlighted were the presence of good immune and virological factors and a stable social environment. Nevertheless, a study from West and Central Africa showed that poor social conditions were not at all a barrier to multi-month dispensing; more patients under a multi-month therapy dispensing kept an undetectable viral load at 24 months [31]. The poor social conditions in Africa are not of the same type as those mentioned in our study (lack of health insurance coverage, access to housing or accommodation, access to rights, resources), but show that in other contexts and conditions, multidrug dispensing is seen as an intervention to increase access to ART to hard-to-reach populations and these elements should be taken into account.

The pharmacists were more cautious: they anticipated more downsides to 90D, with only 5% of pharmacists ready to endorse 90D without any criteria. Besides administrative complexities that could be resolved by ministerial order, 71% of pharmacists anticipated a deficit in monitoring adherence to therapy, drug–drug interactions, and adverse effects (*vs*. 34% of doctors and 10% of PLHIV). This is a compelling point at a time when the average age of PLHIV on care programs in France is progressively rising and comorbidities are becoming more frequent, as pharmacists play a front-line role in HIV care monitoring and support [18]. This issue should lead us to potentially managing the aging population differently, possibly with monthly ART dispensation and close pharmaceutical follow up for certain aging patients. But overall with 90D, patients could be empowered to self-manage. We believe we must facilitate patient self-health care management to get help when it is required, particularly in a well-educated, higher resource setting, and that multidrug dispensing is an important step toward promoting self-management HIV as part of daily-routine life.

This comparative analysis of people on ART (PLHIV and people on PrEP), doctors and pharmacists surveyed on the perceived benefits and limitations of 90D suggested that it would be more convenient for most people on ART and would have no major limitations for many of them. Most people on ART surveyed see the switch from 30-day to 90-day ART dispensing as a good option, whereas doctors and especially pharmacists anticipated a need for advance regulatory groundwork to clarify whether ART drugs can be re-dispensed if doses get lost or in the case of prescriptions going beyond the expiration date. Indeed, the cost of ARVs is high in France, about 700 euros per month or 2000 euros ($2266 USD) per quarter. The remaining issue is that longer dispensing intervals mean longer intervals between opportunities for a patient to get in touch with a pharmacist supporting his or her adherence to therapy and drug–drug interactions. Routine HIV care surveillance is mainly in the hands of HIV-focused pharmacists, who are acutely conscious of this role, as confirmed by this survey. There is a need for pharmacists to be given tailored France-specific guidance on adherence support and 90-day dispensing delivery for people on ART or even for other chronic diseases [32, 33]. Finally, the focus should be placed upstream on health education to encourage self-care management and primary prevention of comorbidities.

## Supporting information

S1 Appendix Presentation letter of the study. French.
(DOCX)

S2 Appendix Participating department identification. French.
(DOCX)

S3 Appendix Questionnaire for PLHIV and people on PrEP. French.
(DOCX)

S4 Appendix Questionnaire for PLHIV and people on PrEP. English.
(DOCX)

S5 Appendix Questionnaire for dotors. French.
(DOCX)

S6 Appendix Questionnaire for doctors. English.
(DOCX)

S7 Appendix Questionnaire for pharamcists. French.
(DOCX)

S8 Appendix Questionnaire for pharmacists. English.
(DOCX)

S9 Appendix Information letter for doctors and phamacists. French.
(DOCX)

S10 Appendix Information letter for PLHIV and people on PrEP. French.
(DOCX)

S11 Appendix Study proposal for pharmacists. French.
(DOC)

S1 Protocol Protocol. French.
(DOCX)
