# Supplementary material for: To what extent do people living with HIV, people on pre-exposure prophylaxis, doctors and pharmacists endorse 90-day dispensing of antiretroviral therapy in France?
Source: PLoS One. 2022 Apr 8;17(4):e0265166. doi: 10.1371/journal.pone.0265166 (PMC8992981; doi:10.1371/journal.pone.0265166)

Mesdames, Messieurs les Présidents, Vice-Présidents et Coordinateurs des COREVIH,

Sous l’impulsion du groupe «médicament/ pharmacien» de la SFLS et des souhaits de nos tutelles, nous vous proposons une nouvelle enquête destinée à connaître «**les souhaits des PVVIH, des personnes sous PrEP, de leur médecin et de leur pharmacien, sur la dispensation trimestrielle des ARVs »** qui sera effectuée **du 12 au 16 octobre 2020.**

En effet, si l’on s’intéresse au 4^ème^ 90 de la cascade de soin, celle de la qualité de vie, celle-ci peut être altérée par les discriminations dont sont victimes les personnes vivant avec le VIH, mais également par une lourdeur de prise en charge héritée des « années sida ». Une des pistes de meilleure qualité de vie pourrait être l’allègement de la dispensation des ARV, actuellement mensuelle, en une dispensation trimestrielle dans certaines situations. De même, depuis la mise à disposition de la prophylaxie pré-exposition (PrEP) en France en 2016, la dispensation est mensuelle. Celle-ci pourrait devenir trimestrielle afin de concourir à une meilleure qualité de vie.

Une autre étude est réalisée par ailleurs et concomitamment pour connaître l’impact économique du conditionnement trimestriel de certains ARVs à l’aide d’une microsimulation entre 2020 et 2025.

Il s’agit de trois courts questionnaires, l’un destiné aux médecins des services participants, l’autre aux PVVIH/personnes sous PrEP reçus en consultation -sans retour dans les dossiers patients-, et le dernier aux pharmaciens à remettre par le PVVIH avec l’ordonnance de ses ARV.

Vous trouverez ci-joint **tous les documents de l’étude**, que nous vous demandons – si vous en êtes d’accord - de diffuser largement auprès de tous les services  qui prennent en charge l’infection VIH sur le territoire de votre COREVIH.

Votre interlocuteur pour cette enquête sera  Emilie GONCALVES : [egoncalves@chu-clermontferrand.fr](mailto:egoncalves@chu-clermontferrand.fr)

En vous remerciant de votre implication,

Très cordialement.

***JACOMET Christine, LANGLOIS Julie,*** ***ZUCMAN David,  BILLAUD Eric,***  ***SIMON Anne,*** ***PUGLIESE Pascal, ARVIEUX Cédric,***

***CERTAIN Agnès, TROUT Hervé, LAURANDIN Bruno, MAAREK René, RAYMOND Isabelle, CHEDORGE Didier,*** ***PUGLIESE-WEHRLEN Sylvia***

COREVIH Auvergne Loire

(COordination REgionale de lutte contre le VIH)

CHU Gabriel Montpied – 7^ème^ HNA

58 rue Montalembert  - 63003 Clermont-Ferrand Cedex 1

Tél. 04 73 75 45 05        Fax 04 73 75 22 79

[www.corevih-auvergne-loire.org](http://www.corevih-auvergne-loire.org/)


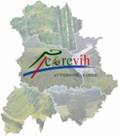

Supplement: S1 Appendix — French. (DOCX) [file pone.0265166.s001.docx]
