# Supplementary material for: To what extent do people living with HIV, people on pre-exposure prophylaxis, doctors and pharmacists endorse 90-day dispensing of antiretroviral therapy in France?
Source: PLoS One. 2022 Apr 8;17(4):e0265166. doi: 10.1371/journal.pone.0265166 (PMC8992981; doi:10.1371/journal.pone.0265166)
Supplement: S2 Appendix — French. (DOCX) [file pone.0265166.s002.docx]

**Enquête une semaine donnée**

**« *Que pensent les PVVIH, les médecins et les pharmaciens de la dispensation trimestrielle des ARV*»**

Semaine du 12 au 16 octobre 2020

**Identification du service participant**

**🞏 CHU 🞏 CHR**

**Nom du service : ______________________________________________________**

**Adresse : ______________________________________________________**

**Code postal : _________________ / Ville : ___________________________**

- **Chef de service :**

**Nom ________________________________Prénom______________________________**

**Adresse mail .......................................................................@.................................................**

**Tel ________________________________**

**Référents du service :**

- **TEC :**

**Nom ________________________________Prénom______________________________**

**Adresse mail .......................................................................@.................................................**

**Tel ________________________________**

- **Médecin :**

**Nom ________________________________Prénom______________________________**

**Adresse mail .......................................................................@.................................................**

**Tel ________________________________**

**🞏 Notre service accepte de participer à l’enquête, une semaine donnée, «***Que pensent les PVVIH, les médecins et les pharmaciens de la dispensation trimestrielle des ARV***». Les correspondants désignés s’engagent à tout mettre en œuvre pour organiser la semaine d’enquête, le recueil et la transmission des données vers l’investigateur principal.**

**🞏 Notre service ne souhaite pas participer à l’enquête.**

**Le responsable du service __________________________**

Cachet du service

**Date : ________/ _________/__________**

**Signature : Signature : ___________________________**

**A FAXER : AU 04 73 75 22 79**

| **Le groupe pilote : adressera un accusé aux référents du service après l’enregistrement des données d’identification, avec attribution d’un code service # # # en 3 chiffres.** |
| --- |

**Enquête une semaine donnée**

**« *Que pensent les PVVIH, les médecins et les pharmaciens de la dispensation trimestrielle des ARV*»**

Semaine du 12 au 16 octobre 2020

**Identification du service participant**

Fiche complétée par le T.E.C. référent du service

**Fiche transmise dans les 8 jours qui suivent la réception de l’enregistrement du service comme participant à l’enquête.**

**Numéro code du service _ _ _ /**

**(cf. Accusé de réception de l’enregistrement du service. Adressé par le groupe de pilotage)**

**Nombre de médecins consultants acceptant l’enquête ___________/**

**Nombre de patients VIH+ habituels le jour de maximum d’activité___________/**

**Nombre de patients sous PrEP habituels le jour de maximum d’activité___________/**

**Nombre des médecins participants. Le TEC attribuera localement un code à chaque médecin**

**1^er^ code _01 _/**

**2eme code _02 _/**

**3eme code _03 _/**

**4eme code _04 _/**

**5eme code _05_/**
