# Supplementary material for: To what extent do people living with HIV, people on pre-exposure prophylaxis, doctors and pharmacists endorse 90-day dispensing of antiretroviral therapy in France?
Source: PLoS One. 2022 Apr 8;17(4):e0265166. doi: 10.1371/journal.pone.0265166 (PMC8992981; doi:10.1371/journal.pone.0265166)
Supplement: S3 Appendix — French. (DOCX) [file pone.0265166.s003.docx]

**Questionnaires pour les patients**

Actuellement, les médicaments antirétroviraux que vous prenez ne peuvent être dispensés par une pharmacie de ville ou hospitalière que mois par mois. La pharmacie peut être autorisée à vous donner plusieurs mois de traitement seulement dans certains cas, notamment dans le cadre d’un départ à l’étranger. A travers ce questionnaire nous souhaitons vous interroger sur le rythme de cette dispensation de traitement en pharmacie.

Vous prenez actuellement des médicaments antirétroviraux dans le cadre de l’infection par le VIH ?

🞏 OUI 🞏 NON

Vous prenez actuellement des médicaments antirétroviraux dans le cadre d’une PrEP (traitement préventif) ?

🞏 OUI 🞏 NON

Si oui, pour l’une ou l’autre, de ces situations, depuis combien de temps ?

- Moins de 1 an
- Entre 1 an et 10 ans
- Plus de 10 ans

Pour ce traitement, vous êtes suivi :

🞏 En ville

🞏 A l’hôpital

🞏 Les deux

Habituellement, vous allez chercher votre traitement :

- Toujours en pharmacie de ville
- Toujours en pharmacie hospitalière
- Indifféremment dans l’une ou l’autre

Concernant votre traitement antirétroviral actuel :

1/ il se présente sous la forme :

- d’un seul comprimé à prendre en une prise dans 1 journée
- de deux comprimés à prendre en une prise dans 1 journée
- de trois comprimés à prendre en une prise dans 1 journée
- de comprimés à prendre plusieurs fois par jour

2/ il vous est dispensé :

- Depuis moins de 6 mois
- Depuis plus de 6 mois

3/vous le prenez :

- Quotidiennement (tous les jours)
- Pas quotidiennement

4/ votre charge virale est

🞏 Indétectable depuis plus de 6 mois

🞏 Indétectable depuis moins de 6 mois

🞏 Détectable

Actuellement les médicaments de cette maladie ne peuvent être dispensés par la pharmacie, que mois par mois, si vous restez sur le territoire.

Seriez-vous intéressé pour avoir 3 mois d’antirétroviraux en une seule fois si le médecin est d’accord ?

🞏 OUI 🞏 NON 🞏 PAS DE PREFERENCE

Pour vous, quels seraient les avantages à avoir une dispensation de médicaments pour 3 mois, en une fois (plusieurs réponses possibles) :

- Plus pratique
- Moins de risques de rupture de traitement en fin de mois
- Plus d’autonomie
- Meilleure qualité de vie
- Plus économique
- Plus de confidentialité
- Autre : …….

Pour vous, quels seraient les inconvénients et les risques en cas de dispensation de médicaments pour 3 mois, en une fois (plusieurs réponses possibles) :

- Risque de complication réglementaire
- Inquiétude sur la dispensation en cas de perte d’un conditionnement de 3 mois
- Sensation d’insécurité et de banalisation de ne voir le pharmacien que tous les 3 mois
- Risque de manque de stock de conditionnements de 3 mois à la pharmacie
- Trop de stock à la maison, manque de confidentialité par rapport aux proches
- Plus cher
- Autre : ….

**Vous êtes :**

- 1. Un homme
  2. Une femme
  3. Une femme trans
  4. Un homme trans
  5. Je ne souhaite pas me définir par le genre

Dans quel département résidez-vous ? …………………..

Vos commentaires et suggestions :

………………………………………………………………………………………………………………………………………………………………………………………………………………………………………………………………………………………………………………………………………………

**Merci d’avoir participé !**
