# Supplementary material for: To what extent do people living with HIV, people on pre-exposure prophylaxis, doctors and pharmacists endorse 90-day dispensing of antiretroviral therapy in France?
Source: PLoS One. 2022 Apr 8;17(4):e0265166. doi: 10.1371/journal.pone.0265166 (PMC8992981; doi:10.1371/journal.pone.0265166)
Supplement: S4 Appendix — English. (DOCX) [file pone.0265166.s004.docx]

Patient # _ _ _ /_ _ / _ _ _ _

Currently, the antiretroviral drugs you take can only be dispensed by a city or hospital pharmacy on a month-to-month basis. The pharmacy may be authorized to give you several months of treatment only in certain cases, in particular in the context of a departure abroad. Through this questionnaire we would like to ask you about the rhythm of this dispensing of treatment in pharmacies.

Are you currently taking antiretroviral drugs for HIV infection?

YES NO

Are you currently taking antiretroviral drugs as part of PrEP (preventive treatment)?

YES NO

If yes, for either of these situations, for how long?

- Less than 1 year

- Between 1 and 10 years

- More than 10 years

For this treatment, you are followed :

In city medical practice

In hospital

Both

Usually, you pick up your treatment :

- Always in a city pharmacy

- Always in a hospital pharmacy

- Either one or the other

Concerning your current antiretroviral treatment:

1/ it is in the form of :

- a single tablet to be taken in one day

- two tablets to be taken in one day

- three tablets to be taken in one dose in one day

- tablets to be taken several times a day

2/ you are dispensed :

- For less than 6 months

- For more than 6 months

3/ you take it :

- Daily (every day)

- Not daily

4/ your viral load is

Undetectable for more than 6 months

Undetectable for less than 6 months

Detectable

At present, medication for this disease can only be dispensed by the pharmacy on a month-to-month basis, if you remain in the country.

Would you be interested in getting 3 months of antiretroviral drugs at once if the doctor agrees?

YES

NO

NO PREFERENCE

For you, what would be the advantages of having 3 months of medication dispensed at once (several answers possible):

- More convenient

- Less risk of treatment breakage at the end of the month

- More autonomy

- Better quality of life

- More economical

- More privacy

- Other: .......

For you, what would be the disadvantages and risks of dispensing medication for 3 months at a time (several answers possible):

- Risk of regulatory complications (special documents to be signed with CPAM/pharmacist...)

- Concern about dispensing in case of loss of a 3-month supply

- Feeling of insecurity and trivialization of seeing the pharmacist only every 3 months

- Risk of lack of stock of 3-month packs at the pharmacy

- Too much stock at home, lack of confidentiality with respect to family and friends

- More expensive

- Other: ....

You are:

Male

A woman

A trans woman

A trans man

I do not wish to define myself by gender

In which department do you live? .......................

Your comments and suggestions :

..........................................................................................................................................................................................................................................................................................................................................................................................

Thank you for your participation! To be returned by mail or fax to 04-73-75-22-79
