# Supplementary material for: To what extent do people living with HIV, people on pre-exposure prophylaxis, doctors and pharmacists endorse 90-day dispensing of antiretroviral therapy in France?
Source: PLoS One. 2022 Apr 8;17(4):e0265166. doi: 10.1371/journal.pone.0265166 (PMC8992981; doi:10.1371/journal.pone.0265166)
Supplement: S5 Appendix — French. (DOCX) [file pone.0265166.s005.docx]

**Questionnaires médecin**

Vous êtes : 🞏 hospitalier CHU 🞏 hospitalier CHG 🞏 libéral

Vous prescrivez des médicaments dans le cadre de l’infection par le VIH et/ou de la PrEP ?

🞏 OUI 🞏 NON

Votre file active :

PVVIH sous ARV : 🞏 Entre 0 et 100

🞏 Entre 100 et 500

🞏 Plus de 500

Personnes sous PrEP :

🞏 Entre 0 et 30

🞏 Entre 30 et 100

🞏 Plus de 100

Actuellement les médicaments antirétroviraux ne peuvent être dispensés par la pharmacie, que mois par mois, si le patient reste sur le territoire.

Pensez-vous que la dispensation d’un conditionnement de 3 mois d’antirétroviraux, en une seule fois au patient, soit envisageable dans certains cas ?

🞏 OUI 🞏 NON 🞏 ne sait pas

Si OUI, dans quelles situations :

- Pas de conditions particulières
- Conditions particulières (plusieurs réponses possibles)
  - Relatives au conditionnement du traitement de 3 mois :
- Quelque soit le traitement (1 ou plusieurs comprimés par jour, 1 prise ou plusieurs prises par jour)
- Uniquement si le traitement est composé d’un seul comprimé par jour
- Uniquement en cas de traitement continu (c'est-à-dire si le traitement VIH n’est pas intermittent (2, 3 ou 4 jours par semaine) ou si la PrEP n’est pas à la demande).
  - Relatives aux conditions immunovirologiques :
- Si le traitement a débuté depuis > 6 mois et si le patient est d’accord
- Si la CV est indétectable depuis > 6 mois et CD4 > 500 et si le patient VIH+ est d’accord
- Si l’observance est bonne, quels que soient les résultats et si le patient est d’accord
- Si le traitement est débuté depuis au moins 6 mois ET la CV est indétectable depuis > 6 mois ET l’observance est bonne et si le patient VIH+ est d’accord.
  - Relatives aux conditions sociales du patient :
- Quelles que soient les conditions sociales du patient
- Si les conditions sociales du patient vous paraissent stables (en terme de couverture sociale, d’hébergement, d’accès aux droits, de ressources)
- A la demande du patient, quels que soient les conditions sociales et les résultats des bilans
- Autres :

Pour vous, les avantages à avoir une dispensation de médicaments pour 3 mois, en une fois pour les patients seront :

| Avantages | absents | Peu importants | importants | Très importants | neutre |
| --- | --- | --- | --- | --- | --- |
| Plus pratique |  |  |  |  |  |
| Moins de risques de rupture de traitement en fin de mois |  |  |  |  |  |
| Plus d’autonomie |  |  |  |  |  |
| Meilleure confidentialité |  |  |  |  |  |
| Meilleure qualité de vie |  |  |  |  |  |
| Plus économique |  |  |  |  |  |
| Autre, définissez |  |  |  |  |  |

Pour vous, les inconvénients et les risques en cas de dispensation de médicaments pour 3 mois, en une fois pour les patients seront :

| Risques/inconvénients | absents | peu importants | importants | très importants | systématiques |
| --- | --- | --- | --- | --- | --- |
| Complication administrative (en termes de dispensation gratuite et/ou remboursement) |  |  |  |  |  |
| Non dispensation en cas de perte d’un conditionnement de 3 mois |  |  |  |  |  |
| Défaut de suivi sur l’observance, les interactions médicamenteuses et les effets indésirables par le pharmacien |  |  |  |  |  |
| Difficultés de dispensation en cas de traitement intermittent (type 4D chez les PVBVIH ou à la demande en cas de PrEP) |  |  |  |  |  |
| Nécessité d’anticipation du patient (car pas de stock possible) |  |  |  |  |  |
| Plus cher |  |  |  |  |  |
| Autre, définissez |  |  |  |  |  |

Vos commentaires et suggestions :

………………………………………………………………………………………………………………………………………………………………………………………………………………………………………………………………………………………………………….…………………………………..

Merci d’avoir participé !
