# Supplementary material for: To what extent do people living with HIV, people on pre-exposure prophylaxis, doctors and pharmacists endorse 90-day dispensing of antiretroviral therapy in France?
Source: PLoS One. 2022 Apr 8;17(4):e0265166. doi: 10.1371/journal.pone.0265166 (PMC8992981; doi:10.1371/journal.pone.0265166)
Supplement: S6 Appendix — English. (DOCX) [file pone.0265166.s006.docx]

**Physician N° _ _ _ /_ _ /**

You work : in universitary hospital

in non universitary hospital

private practice

Do you prescribe medication for HIV infection and/or PrEP?

YES NO

Your active file :

PLWH on ARV: Between 0 and 100

Between 100 and 500

More than 500

People on PrEP :

Between 0 and 30

Between 30 and 100

More than 100

Currently, antiretroviral drugs can only be dispensed by the pharmacy on a month-to-month basis, if the patient remains in the country.

Do you think that dispensing a 3-month supply of antiretroviral drugs to the patient in one go is feasible in some cases?

YES

NO

don't know

If YES, in what situations?

- No specific conditions

- Special conditions (several answers possible)

o Related to the 3-month treatment package:

- Whatever the treatment (1 or more tablets per day, 1 shot or multiple shots per day)

- Only if the treatment consists of one tablet per day

- Only if continuous treatment (i.e., if HIV treatment is not intermittent (2, 3, or 4 days per week) or if PrEP is not on demand).

o Related to immunovirological conditions:

- If treatment started > 6 months ago and patient agrees

- If CV has been undetectable for > 6 months and CD4 > 500 and if the HIV+ patient agrees

- If compliance is good, regardless of results and if the patient agrees

- If treatment has been started for at least 6 months AND CV has been undetectable for > 6 months AND adherence is good and if the HIV+ patient agrees.

o Related to the patient's social conditions:

- Regardless of the patient's social conditions

- If the patient's social conditions seem stable to you (in terms of social coverage, accommodation, access to rights, resources)

- At the patient's request, regardless of the social conditions and the results of the assessments

Other: define….

For you, the benefits of having a 3-month dispensation of medication, at one time for patients, will be:

| Benefits : | None | Not important | Important | Very important | neutral |
| --- | --- | --- | --- | --- | --- |
| More convenient |  |  |  |  |  |
| Less risk of treatment rupture at the end of the month |  |  |  |  |  |
| More autonomy |  |  |  |  |  |
| Better confidentiality |  |  |  |  |  |
| Better quality of life  More economical |  |  |  |  |  |
| Other, please define |  |  |  |  |  |

For you, the disadvantages and risks of dispensing medications for 3 months at a time for patients will be:

| Risks/disadvantages | none | not very important | important | very important | neutral |
| --- | --- | --- | --- | --- | --- |
| Administrative complication  (with insurance) |  |  |  |  |  |
| Non-dispensing in case of loss of a 3-month package |  |  |  |  |  |
| Lack of follow-up on compliance, drug interactions Dispensing difficulties in case of intermittent treatment (4D type in PLHIV or on demand in case of PrEP) and side effects by the pharmacist |  |  |  |  |  |
| Need for patient anticipation (as no stock is available) |  |  |  |  |  |
| More expensive |  |  |  |  |  |
| Other, please define |  |  |  |  |  |

Your comments and suggestions :

..........................................................................................................................................................................................................................................................................................................................................................................................

Thank you for your participation! To be returned by mail or fax to 04-73-75-22-79
