# Supplementary material for: To what extent do people living with HIV, people on pre-exposure prophylaxis, doctors and pharmacists endorse 90-day dispensing of antiretroviral therapy in France?
Source: PLoS One. 2022 Apr 8;17(4):e0265166. doi: 10.1371/journal.pone.0265166 (PMC8992981; doi:10.1371/journal.pone.0265166)
Supplement: S8 Appendix — English. (DOCX) [file pone.0265166.s008.docx]

**Pharmacist No. _ _ _ /_ _ /_ _ /P**

You work: in university hospital

In non-university hospital

private practice

Department of practice: .............

Do you dispense medication for HIV infection and/or PrEP?

YES NO

Your active file:

PLWH on ARV: Between 0 and 2

Between 2 and 30

Between 30 and 100

More than 100

People on PrEP :

Between 0 and 2

Between 2 and 30

More than 30

Currently, antiretroviral drugs can only be dispensed by the pharmacy on a month-to-month basis, if the patient remains in the country.

Do you think that dispensing a 3-month supply of antiretroviral drugs to the patient at once is feasible in some cases?

YES

NO

Don't know

If YES, in what situations?

- No specific conditions

- Only if you have already given ARVs to this patient

- Special conditions (several answers possible)

o Related to the presentation of the treatment:

- Whatever the treatment (1 or more tablets per day, 1 dose or several doses per day)

- Only if the treatment consists of one tablet per day

- Only if continuous treatment (i.e., if HIV treatment is not intermittent (2, 3, or 4 days per week) or if PrEP is not on demand)

o Related to immunovirological conditions:

- If treatment started > 6 months ago and patient and physician agree

- If CV has been undetectable for > 6 months and CD4 > 500 and if the HIV+ patient and the physician agree

- If compliance is good, regardless of results, and if the patient and physician agree

- If treatment has been started for at least 6 months AND CV has been undetectable for > 6 months AND adherence is good and if the HIV+ patient and physician agree

o Related to the patient's social conditions:

- Regardless of the patient's social conditions

- If the patient's social conditions seem stable (in terms of social coverage, housing, access to rights, resources)

- At the patient's request, regardless of the social conditions and the results of the assessments

- Other :

For you, the benefits of having a 3-month, one-time medication dispensation for patients will be:

| Benefits : | None | Not important | Important | Very important | neutral |
| --- | --- | --- | --- | --- | --- |
| More convenient |  |  |  |  |  |
| Less risk of treatment rupture at the end of the month |  |  |  |  |  |
| More autonomy |  |  |  |  |  |
| Better confidentiality |  |  |  |  |  |
| Better quality of life  More economical |  |  |  |  |  |
| Other, please define |  |  |  |  |  |

For you, the disadvantages and risks of dispensing medications for 3 months at a time for patients will be :

| Risks/disadvantages | none | not very important | important | very important | neutral |
| --- | --- | --- | --- | --- | --- |
| Administrative complication  (with insurance) |  |  |  |  |  |
| Non-dispensing in case of loss of a 3-month package |  |  |  |  |  |
| Lack of follow-up on compliance, drug interactions Dispensing difficulties in case of intermittent treatment (4D type in PLHIV or on demand in case of PrEP) and side effects by the pharmacist |  |  |  |  |  |
| Need for patient anticipation (as no stock is available) |  |  |  |  |  |
| More expensive |  |  |  |  |  |
| Other, please define |  |  |  |  |  |

Your comments and suggestions :

..........................................................................................................................................................................................................................................................................................................................................................................................

Thank you for your participation! To be returned by fax to 04-73-75-22-79
