# Supplementary material for: To what extent do people living with HIV, people on pre-exposure prophylaxis, doctors and pharmacists endorse 90-day dispensing of antiretroviral therapy in France?
Source: PLoS One. 2022 Apr 8;17(4):e0265166. doi: 10.1371/journal.pone.0265166 (PMC8992981; doi:10.1371/journal.pone.0265166)
Supplement: S9 Appendix — French. (DOCX) [file pone.0265166.s009.docx]

**Lettre d’information**

Le CHU de Clermont-FD vous propose de participer à une enquête destinée à connaître votre avis sur la dispensation trimestrielle des ARV.

Il s’agit pour vous de répondre à un questionnaire sur votre avis vis-à-vis des traitements antirétroviraux.

Votre participation à cette étude est entièrement libre et volontaire. Ce questionnaire est « anonyme » (votre nom n’apparaitra pas) et identifié seulement par un code. La durée de remplissage du questionnaire est d’environ 2 à 5 minutes.

Conformément aux dispositions de la loi relative à l’informatique aux fichiers et aux libertés, vous pouvez refuser de participer à cette enquête, et vous pouvez à tout moment vous retirer de cette recherche, sans préjudice.

Si vous acceptez, vous remplirez ce questionnaire et le remettrez à la personne chargée de l’étude. Les seuls destinataires des données sont des personnels du CHU impliqués dans l’étude (soumis au secret professionnel).

Conformément au RGPD et à la loi Informatique et Libertés du 6 janvier 1978 modifiée (par la [loi n° 2018-493 du 20 juin 2018](https://www.legifrance.gouv.fr/eli/loi/2018/6/20/JUSC1732261L/jo/texte)), vous avez le droit d’avoir communication des données vous concernant et le droit de demander éventuellement la limitation ou l’effacement de ces données si vous décider d’arrêter votre participation à l’étude.

Vous avez également la possibilité de vérifier l’exactitude des informations que vous aurez fournies et la possibilité de demander éventuellement leur correction. Ces droits d’accès, de rectification, et de limitation du traitement de vos données personnelles pourront s’exercer à tout moment en adressant une demande écrite à l’investigateur principal de la recherche. Vos données seront conservées 2 ans après la fin de l’étude.

Obtention d’informations complémentaires :

Si vous le souhaitez, vous pourrez durant toute la durée de l’étude contacter les responsables pour obtenir des précisions ou des informations complémentaires.

- Pour toute question concernant l’étude, retrait de consentement, ou pour exercer vos droits concernant vos données (accès, rectification, etc…) : vous pouvez contacter L’investigateur principal de la recherche : Dr JACOMET Christine CHU de Clermont-FD 58 rue Montalembert 63003 Clermont-Ferrand

🖂 cjacomet@chu-clermontferrand.fr

- Pour toute question relative à la protection de vos données personnelles : vous pouvez contacter le délégué à la protection des données, Michel Rubio, CHU de Clermont-FD 58 rue Montalembert 63003 Clermont-Ferrand

🖂 [dpd@chu-clermontferrand.fr](mailto:dpd@chu-clermontferrand.fr)

La base légale de ce traitement est l’intérêt légitime du CHU de Clermont-Ferrand, en tant qu’établissement de santé Hospitalo-Universitaire à vocation de recherche, à mettre en œuvre le traitement de données médicales à des fins de recherche scientifique (article 9.2 du RGPD).

Si vous estimez, après nous avoir contactés, que vos droits Informatique et Libertés ne sont pas respectés, vous pouvez également porter une réclamation auprès de la CNIL: <https://www.cnil.fr/fr/webform/adresser-une-plainte/>).

De même, vous pouvez accéder directement ou par l’intermédiaire d’un médecin de votre choix à l’ensemble de vos données médicales en application des dispositions de l’article L 1111-7 du Code de la Santé Publique.
