# Supplementary material for: To what extent do people living with HIV, people on pre-exposure prophylaxis, doctors and pharmacists endorse 90-day dispensing of antiretroviral therapy in France?
Source: PLoS One. 2022 Apr 8;17(4):e0265166. doi: 10.1371/journal.pone.0265166 (PMC8992981; doi:10.1371/journal.pone.0265166)
Supplement: S11 Appendix — French. (DOC) [file pone.0265166.s011.doc]

Clermont Ferrand

Le 19/06/2020

Cher confrère, Cher collègue,

Le groupe Médicament/Pharmaciens de la Société Française de Lutte contre le Sida (SFLS)[[1]](#footnote-2) vous propose de participer à une enquête destinée à connaitre les souhaits des personnes concernées par le VIH (VIH+ et sous PrEP), des médecins et des pharmaciens en ce qui concerne une dispensation trimestrielle éventuelle des antirétroviraux.

Un patient vient de vous remettre cette lettre avec une note d’information, ainsi qu’un questionnaire.

Il s’agira pour vous

1/ de lire la lettre et si souhaité, de répondre au questionnaire vous concernant

2/ de renvoyer par fax le questionnaire rempli au n° **04 73 75 22 79**

Il n’y a pas d’autre chose à faire : pas de retour sur le dossier pharmaceutique. La seule contrainte est d’être exhaustif.

Merci !

Bien à vous.

Docteur C. JACOMET et le Groupe M/P[[2]](#footnote-3) SFLS

1. http://www.sfls.aei.fr/Commission-pharmaciens-medicaments [↑](#footnote-ref-2)
2. Groupe M/P SFLS :Jean-Félix Albrecht, Anne Armand, Philippe Arsac, Eric Billaud, Emmanuelle Boschetti, Pierre Bouttaz, Laurence Boyer, Agnès Certain, Didier Chedorge, Louis Do, Agnès Gautheret, Christine Jacomet, Julie Langlois, Bruno Laurandin, René Maarek, Alexandra Muzard, Isabelle Raymond, Hervé Trout, Sylvia Wehrlen, David Zucman [↑](#footnote-ref-3)
