# Supplementary material for: To what extent do people living with HIV, people on pre-exposure prophylaxis, doctors and pharmacists endorse 90-day dispensing of antiretroviral therapy in France?
Source: PLoS One. 2022 Apr 8;17(4):e0265166. doi: 10.1371/journal.pone.0265166 (PMC8992981; doi:10.1371/journal.pone.0265166)
Supplement: S1 Protocol — French. (DOCX) [file pone.0265166.s012.docx]

**« Que pensent les PVVIH, les médecins et les pharmaciens de la dispensation trimestrielle des antirétoviraux »**

**Enquête une semaine donnée. (12-16 Oct 2020)**

***Investigateur coordonnateurs***

***JACOMET Christine*** [***cjacomet@chu-clermontferrand.fr***](mailto:cjacomet@chu-clermontferrand.fr)

***LANGLOIS Julie*** [***jlanglois.pharma@gmail.com***](mailto:jlanglois.pharma@gmail.com)

***Investigateurs cliniciens***

***ZUCMAN David*** [***d.zucman@hopital-foch.org***](mailto:d.zucman@hopital-foch.org)

***BILLAUD Eric*** [***eric.billaud@chu-nantes.fr***](mailto:eric.billaud@chu-nantes.fr)

***SIMON Anne*** [***anne.simon@aphp.fr***](mailto:anne.simon@aphp.fr)

***PUGLIESE Pascal*** [***pugliese.p@chu-nice.fr***](mailto:pugliese.p@chu-nice.fr)

***ARVIEUX Cédric cedric.arvieux@chu-rennes.fr***

***Investigateurs pharmaciens***

***CERTAIN Agnès*** [***agnes.certain@orange.fr***](mailto:agnes.certain@orange.fr)

***TROUT Hervé*** [***herve.trout@aphp.fr***](mailto:herve.trout@aphp.fr)

***LAURANDIN Bruno*** [***contact@pharmaciedeschenes.fr***](mailto:contact@pharmaciedeschenes.fr)

***MAAREK René*** [***rene_maarek@hotmail.com***](mailto:rene_maarek@hotmail.com)

***RAYMOND Isabelle*** [***isabelle.raymond@chu-bordeaux.fr***](mailto:isabelle.raymond@chu-bordeaux.fr)

***CHEDORGE Didier*** [***Didierchedorge@hotmail.com***](mailto:Didierchedorge@hotmail.com)

***PUGLIESE-WEHRLEN Sylvia pugliese.s@chu-nice.fr***

**Méthodologie**

**LAMBERT Céline** [***clambert@chu-clermontferrand.fr***](mailto:clambert@chu-clermontferrand.fr)

**GONCALVES Emilie** [***egoncalves@chu-clermontferrand.fr***](mailto:egoncalves@chu-clermontferrand.fr)

**Promoteurs  CHU Clermont Ferrand et SFLS**

**Introduction**

En 2017, lors de la dernière évaluation nationale de la cascade de prise en charge de l’infection VIH en France, dont l’objectif OMS était d’accéder à 90% de personnes dépistées, 90% de personnes sous traitement et 90% de personnes sous traitement ayant une charge virale indétectable (90/90/90) il s’est avéré que 97% de patients sous antirétroviraux (ARV) pris en charge dans le système de soins avaient bien une charge virale indétectable. Le « troisième 90 » est donc dépassé. Cette cascade n’évalue pas la qualité de vie des PVVIH sous ARV. Celle-ci peut être altérée par les discriminations dont sont victimes les personnes vivant avec le VIH, mais également par une lourdeur de prise en charge héritée des « années sida ». Une des pistes de meilleure qualité de vie pourrait être l’allègement de la dispensation des ARV, actuellement mensuelle, en une dispensation trimestrielle dans certaines situations.

De même, depuis la mise à disposition de la prophylaxie pré-exposition (PrEP) en France en 2016, la dispensation est mensuelle. Celle-ci pourrait devenir trimestrielle, en particulier chez ceux-là prenant au long cours, afin de concourir à une meilleure qualité de vie.

Cependant, nous ne disposons pas à l’heure actuelle d’éléments objectifs sur l’ampleur de la demande tout en observant que certains patients expriment ce souhait en mettant en avant l’avantage pratique qu’il y aurait à n’avoir plus que 4 dispensations d’ARV par an.

Les aspects réglementaires sont à prendre en considération. En effet, actuellement, la dispensation est mensuelle (28 ou 30 jours), sauf en cas de départs à l’étranger, avec à l’appui une réglementation particulière (accord de la Caisse du patient, mention du prescripteur, variabilité selon les CPAM ; cf Billaud E . et al, JNI, 2018)

Pour d’autres médicaments, dans le cadre des maladies chroniques, une dispensation pour 3 mois est possible s’il existe des conditionnements de 3 mois. Quatre pathologies sont concernées : diabète, HTA, hypercholestérolémie, ostéoporose. Les contraceptifs ont aussi des présentations trimestrielles. Ces conditionnements génèrent des économies substantielles.

Dans le cas des ARV, plusieurs éléments sont à considérer. Tout d’abord, la stabilité clinique et virologique du patient : il ne semble pas raisonnable de dispenser trimestriellement les antirétroviraux les premiers six mois, ou après une modification alors que la tolérance et l’efficacité n’ont pas encore été évaluées. Il faudra aussi prendre en compte les défauts éventuels d'observance au sein des populations vulnérables, le coût en cas de modification thérapeutique pour mauvaise tolérance, toxicité ou autre, et l’éventuelle perte ou médicaments abîmés. Dans tous ces cas, la balance bénéfice/ risque financier sera en défaveur de la dispensation trimestrielle.

Concernant la Caisse Nationale d’Assurance Maladie, et selon le texte ci-dessus cité, seuls les conditionnements de 3 mois par les firmes pourraient être remboursés. Par contre, la dispensation de 3 mois avec les boîtages mensuels actuels nécessiteraient l’accord de la Caisse, patient par patient (sachant que chaque caisse fonctionne différemment ; (cf ci-dessus disparité pour dispensation en cas de départ à l’étranger) ; de ce fait, cela créerait des inégalités entre patients, selon la zone/région où ils demeurent. Les firmes/laboratoires devront donc aussi être partants.

Du côté du pharmacien, l’investissement devra être plus important. Les stocks pourraient en être diminués, entrainant une commande par patient uniquement, sauf pour les pharmacies qui peuvent se permettre un peu de stock sachant qu’il leur faudrait un échantillonnage important (car il y a maintenant de nombreux combinaisons de trithérapies et de bithérapies commercialisés).

Et les firmes, seraient-elles prêtes à faire des conditionnements de 3 mois ?

Il ressort de cette brève analyse, une difficulté de mesurer les besoins et un certain nombre d’obstacles réglementaires, financiers, techniques, logistiques, industriels.

**Objectif principal**

Recueillir les perceptions sur les avantages et les inconvénients d’une dispensation de 3 mois d’ARV en une fois du point de vue de la personne vivant avec le VIH, de la personne sous PrEP, du pharmacien et du médecin prescripteur.

**Objectifs secondaires**

- Quels sont les profils de ces patients, du médecin et du pharmacien ?
- Quels liens entre les 3 catégories de répondant peuvent-ils être mis en évidence ?

**Méthode**

Enquête nationale une semaine donnée

3 volets

- Volet patient : Critères d’inclusion ; patient VIH+ sous traitement ARV depuis > 6 mois et patient sous PrEP depuis > 6 mois. Critères d’exclusion : mineur, sous tutelle
- Volet médecin : tout médecin recevant des PVVIH et/ou personne sous PrEP la semaine de l’enquête
- Volet pharmacien : tout pharmacien auprès duquel la personne se rend suite à la consultation pour la dispensation de ses ARV.

**Critères d’évaluation**

Analyse globale et par type de traitement (combos en 1 cp par jour vs les autres)

% patients VIH+ préférant une dispensation de 3 mois en une fois.

% patients sous PrEP préférant une dispensation de 3 mois en une fois.

% médecins préférant une dispensation de 3 mois en une fois.

% pharmaciens préférant une dispensation de 3 mois en une fois.

**Déroulement de l’enquête**

Semaine du 12 octobre au 16 octobre 2020,

- Le médecin appartenant à un service acceptant l’étude lit la lettre d’information puis complète le questionnaire papier médecin et le remet au TEC du service
- le patient qui se rend en consultation hospitalière, se voit aussi proposer l’enquête- PVVIH par son médecin. Il lit la lettre d’information, remplit le questionnaire « anonyme » et la non opposition sur site et les remettent au TEC du service
- Lorsque le patient se rend à la pharmacie de dispensation, il remet au pharmacien la lettre d’information, et le pharmacien remplit le questionnaire et l’envoie par fax au TEC du service concerné.
- Les questionnaires sont identifiés comme suit : /---/--/--/--/

---/ numéro de centre à trois chiffres

--/ Numéro d’ordre médecin (stop questionnaire médecin)

--/ Numéro d’ordre patient (stop questionnaire patient)

--/ Numéro d’ordre pharmacien

Le patient peut être aidé dans la compréhension ou la réponse aux questions si besoin.

Il n’y a aucun recours au dossier médical. Il n’y a aucune liste de patients préétablie.

**Organisation globale**

**1^er^ temps : identification des services participants**

Les courriers de proposition d’étude sont envoyés dans les sites de prise en charge via

- les présidents de Corevih, leurs secrétaires, coordinateurs et TEC COREVIH

- les CHU et les CHG prenant en charge les PVVIH

- les pharmacies hospitalières

- les pharmacies d'officines 'sentinelles' identifiées par la SFLS (dans le cadre des formations autotests VIH, enquêtes précédentes)

**2^ème^ temps : envoi du protocole de l’étude et de l’enquête ainsi que la lettre d’information aux centres potentiellement participants.** Ces centres ont préalablement fait l’objet d’un recueil des coordonnées du centre par le CHU de Clermont Ferrand auprès de la CNIL.

**3^ème^ temps : envoi à chaque centre du nombre de questionnaires requis avec une enveloppe réponse (services hospitaliers) ou numéro de fax (officines).**

Ceux ayant accepté l’enquête reçoivent les formulaires à distribuer aux patients et des enveloppes pré-timbrées pour retour.

Il n’y a aucune liste de patients préparée dans un service donné.

Lors de la semaine d’enquêtes tous les patients sont informés lors des consultations / dispensations de la réalisation de celle-ci et répondent ou non selon leur volonté.

Les médecins remettent le questionnaire au TEC. Les patients remettent le questionnaire au médecin ou TEC. Ceux-ci le renvoient par courrier à l’aide de l’enveloppe pré-timbrée au centre de Clermont-Ferrand. Les pharmaciens renvoient le questionnaire par fax.

**4^ème^ temps**

Retour des questionnaires dans la semaine qui suit celle de l’enquête

**Considérations statistiques**

Calcul du nombre de sujets

Ce type d’enquête, menée sur un échantillon, doit permettre de généraliser les résultats à l'ensemble de la population ciblée. Aussi, la taille de l’échantillon est déterminante ; plus l’échantillon est important, plus la généralisation sera fiable.

Dans ce type de calcul d’effectif, on se fixe donc, en plus de la proportion escomptée ((i) taux de patients vivant avec le VIH / sous PrEP estimant que la dispensation trimestrielle est une amélioration de la qualité de vie (ii) taux de médecins et taux de pharmaciens acceptant la dispensation trimestrielle des ARV), une marge d’erreur sur l’estimation (fiabilité de l’échantillon).

Ainsi (cf. figure ci-dessous), pour une proportion de 50%, un minimum de n=600 patients permettra d’avoir une marge d’erreur de 4%, autrement dit permettra d’extrapoler les proportions issues de l’enquête avec 5% de risque (erreur α ou seuil de confiance) de se tromper de ± 4%.


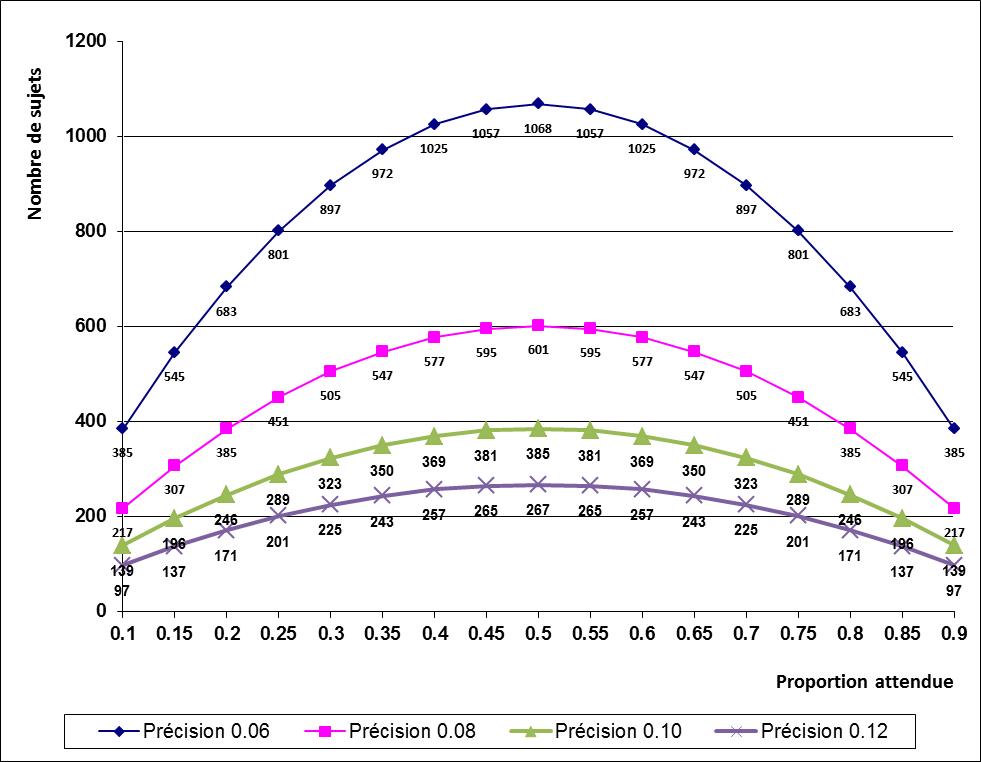


Analyses

Toutes les analyses seront réalisées avec le logiciel Stata (version 13, StataCorp, College Station, Texas). La population sera décrite par des effectifs et pourcentages associés pour les variables catégorielles et par des moyennes ± écart-type ou médiane [intervalle interquartile] pour les variables quantitatives, au regard de leur distribution statistique (normalité étudiée par le test de Shapiro-Wilk).

Le taux de patients vivant avec le VIH, de médecins et de pharmaciens préférant la dispensation trimestrielle seront présentés avec un intervalle de confiance à 95%.

S’agissant d’une étude à visée exploratoire, des analyses multidimensionnelles seront également proposées dans le but de déterminer d’éventuels profils des patients notamment par le biais d’analyses multidimensionnelles factorielles de données mixtes (AFDM) permettant d’analyser en tant qu’éléments actifs des variables qualitatives et quantitatives. En effet, la méthodologie usuelle consiste à transformer les variables quantitatives en paramètres de nature catégorielle en les catégorisant en classes afin de soumettre, *in fine*, ces nouvelles variables ainsi que les variables qualitatives à une analyse exploratoire des correspondances multiples (ACM). Cette méthodologie est relativement facile à mettre en œuvre et peut être utilisée lorsque les individus sont assez nombreux (>100). Sinon, l’ACM donne des résultats peu stables. Aussi, il est intéressant de conserver, par le biais de méthodes telles que l’AFDM, telles quelles les variables quantitatives dans deux situations : (1) lorsque le nombre de variables de nature qualitative est très petit comparativement à celui des variables quantitatives et (2) lorsque le nombre d’individus est faible.

Ces analyses seront complétées par des tests statistiques usuels (par exemple (i) dans le but de comparer les patients estimant un bénéfice de la dispensation trimestrielle (ii) afin de comparer les médecins  et les pharmaciens à savoir : 1) test t de Student ou test de Mann-Whitney si conditions du t-test ne sont pas respectées (homoscédasticité vérifiée par le test de Fisher-Snedecor et normalité par le test de Shapiro-Wilk) pour les variables de nature quantitative et 2) test du Chi2 ou test exact de Fisher le cas échéant pour les variables catégorielles. Si p<0.05, les différences testées seront considérées comme significatives. Des analyses multivariées de type régression logistique (pour variables dépendantes dichotomiques) seront proposées en considérant les covariables au regard des résultats d’analyse univariée et de leur pertinence clinique (dont genre, âge, durée de maladie, durée de mise sous traitement).

Un grand nombre d’analyses étant de nature exploratoire, elles pourraient manquer de puissance statistique. Comme discuté par Feise en 2002 (Feise RJ. *Do multiple outcome measures require p-value adjustment? BMC Medical Research Methodology* 2002, 2:8), l’ajustement du risque d’erreur de 1^ière^ espèce ne sera pas proposé systématiquement, mais au cas par cas au vue des considérations cliniques et non uniquement statistiques.

**Calendrier**

**Mai/juin :** Écriture des documents et mise à jour des listes de diffusion

- Enquête
- Questionnaire patient
- Questionnaire médecin
- Questionnaire pharmacien
- Note d’informations
- Identification des centres
- Avis éthique
- Respect RGPD, procédure MR004

**Juillet /aout 2020 :** Proposition de l’enquête et définition des centres participants

Avis CNIL : liste des services participants recueillis dans le fichier CIL du CHU de Clermont Ferrand.

**Septembre 2020 :** Envoi des documents aux centres

**Octobre 2020 :** Enquête et retour des questionnaires

**Novembre 2020** : Saisie des données

**Décembre 2020** : Premiers résultats diffusés aux membres du groupe
